# In vivo effect of opticin deficiency in cartilage in a surgically induced mouse model of osteoarthritis

**DOI:** 10.1038/s41598-017-18047-w

**Published:** 2018-01-11

**Authors:** Aina Farrán, Gladys Valverde-Franco, Laura Tío, Bertrand Lussier, Hassan Fahmi, Jean-Pierre Pelletier, Paul N. Bishop, Jordi Monfort, Johanne Martel-Pelletier

**Affiliations:** 1 Osteoarthritis Research Unit, University of Montreal Hospital Research Centre (CRCHUM), Montreal, Quebec, Canada; 2 Inflammation and Cartilage Cellular Research Group, IMIM (Hospital del Mar Medical Research Institute) Rheumatology Department, Parc de Salut Mar, Hospital del Mar, Barcelona, Spain; 3 Faculty of Veterinary Medicine, Clinical Sciences, University of Montreal, Saint-Hyacinthe, Quebec, Canada; 4 Manchester Royal Eye Hospital, Central Manchester University Hospitals NHS Foundation Trust, Manchester Academic Health Science Centre, Manchester, United Kingdom; 5 School of Biological Sciences, Faculty of Biology, Medicine and Health, University of Manchester, Manchester, United Kingdom; 6 Rheumatology Department, Hospital del Mar, Barcelona, Spain

## Abstract

The SLRP opticin (OPTC) has been demonstrated to be produced and degraded in osteoarthritic (OA) human cartilage. Here, we investigated the *in vivo* effect of OPTC deficiency in OA cartilage. OA was induced in 10-week-old *Optc* ^−/−^ and *Optc* ^+/+^ mice. Ten weeks post-surgery, cartilage was processed for histology and immunohistochemistry. SLRP expression was determined in non-operated mouse cartilage. OA *Optc* ^−/−^ demonstrated significant protection against cartilage degradation. Data revealed that in non-operated *Optc* ^−/−^ cartilage, expression of SLRPs lumican and epiphycan was up-regulated at day 3 and in 10-week-olds (p ≤ 0.039), and fibromodulin down-regulated in 10-week-olds (p = 0.001). Immunohistochemistry of OA mice showed a similar pattern. In OA *Optc* ^−/−^ cartilage, markers of degradation and complement factors were all down-regulated (p ≤ 0.038). In OA *Optc* ^−/−^ cartilage, collagen fibers were thinner and better organized (p = 0.038) than in OA *Optc* ^+/+^ cartilage. The protective effect of OPTC deficiency during OA results from an overexpression of lumican and epiphycan, known to bind and protect collagen fibers, and a decrease in fibromodulin, contributing to a reduction in the complement activation/inflammatory process. This work suggests that the evaluation of the composition of the different SLRPs in OA cartilage could be applied as a new tool for OA prognosis classification.

## Introduction

Articular cartilage degeneration is the main feature of arthritic joint diseases such as osteoarthritis (OA). The process involves the degradation of extracellular matrix components including the major macromolecules type II collagen and proteoglycans. Proteoglycans are heavily glycosylated proteins that can be divided into large hyaluronan-binding proteoglycans like aggrecan, and small proteoglycans including the small leucine-rich repeat protein (SLRP) superfamily^1^. The latter is divided into five classes (class I-V) of structurally and genetically related molecules^2,3^. Some members of this family have emerged as being capable of binding and regulating collagen fibril assembly and growth^4^, protecting the collagen fibers by surface coating avoiding the access of catabolic factors including proteases^5,6^, while others have been involved in collagen mineralization^7^, and complement regulation^8,9^.

Although not all the functions of the SLRPs have yet been elucidated, several studies demonstrate that the interruption of the normal balance through altered expression of some of them can influence the tissue properties^10–12^. Studies have described the rescue and/or compensation mechanisms by SLRPs when a single member is lacking or modified^13–16^, suggesting a role of the SLRPs in the regulation of the extracellular matrix architecture.

One of the SLRPs, opticin (OPTC; class III), first found associated with vitreous humor collagen fibrils in the eye^17,18^, was further found to be of interest in the human OA process^19,20^. Hence, our group was the first to demonstrate the OPTC expression/production in human articular cartilage and its degradation during OA^19,20^. Moreover, in addition to the chondrocyte, OPTC is also produced by other articular cells: synoviocytes and osteoblasts^19^, and in other tissues: ligaments and skin^17^. OPTC can also be digested by several proteases implicated in OA development^20^.

However, the *in vivo* effect of the absence of OPTC in OA cartilage remains to be clarified. We hypothesized that *in vivo* lack of OPTC would be detrimental to the articular cartilage structure. To this end, we investigated the effect of OPTC deficiency in OA using a mouse model.

## Materials and Methods

### Mice

We used a transgenic mouse in which the *Optc* gene has been deleted using a targeting vector that encompasses exon 1 to exon 4^21^. For further details see Supplementary Material, Materials and Methods and Supplementary Material Fig. S1. All procedures involving animals were performed according to regulations of the Canadian Council on Animal Care and were approved by the Animal Care Committee of the University of Montreal Hospital Centre. All mice were maintained under a 12-hour light/dark cycle. Food and water were available *ad libitum*.

Mouse weight and length were measured on day 5 (P05) and day 16 (P16).

### Surgically Induced OA Mouse Model

OA was surgically induced in 10-week-old male mice (*Optc* ^+/+^ and *Optc* ^−/−^) for 10 weeks by destabilization of the medial meniscus (DMM) of the right knee as described^22,23^. For further details see Supplementary Material, Materials and Methods.

### Evaluation of knee joint swelling

The knee diameter was measured in the mediolateral plane at baseline (day 0) and every 3 days, for 15 days, using a digital caliper (model 2071M; Mitutoyo), as described^24^.

### Extraction of total RNA, reverse transcriptase (RT) PCR, and gene expression determination

Articular cartilage from the knees of P03 and 10-week-old *Optc* ^+/+^ and *Optc* ^−/−^ mice was processed for RNA extraction. Femoral and humeral heads were carefully dissected and placed in TRIzol (Invitrogen, Carlsbad, CA, USA). Tissue was homogenized with a Polytron homogenizer (Kinematica, Bohemia, NY, USA) and total RNA was extracted according to the manufacturer’s specifications and treated with the RapidOut DNA Removal Kit (Thermo Fisher, Waltham, MA, USA) to ensure complete removal of chromosomal DNA. This extraction method yields undegraded RNA as observed by agarose/SYBR Safe nucleic acid stain (Invitrogen) gel electrophoresis and reproducible C_t_ values of the housekeeping gene. Purity and quantitation of total RNA were assessed with NanoDrop (Thermo Fisher).

The RT reactions were primed with random hexamer primers and messenger RNA (mRNA) levels were quantified by real-time PCR (qPCR), performed in the GeneAmp 5700 Sequence Detection System (Applied Biosystems, Foster City, CA, USA) with QuantiTect SYBR Green PCR Master Mix (Qiagen, Valencia, CA, USA), and used according to the manufacturer’s specifications. To evaluate the effect of *Optc* ^−/−^, the expression level of *Optc* ^+/+^ studied genes was assigned an arbitrary value of 1, and *Optc* ^−/−^ cartilage expression evaluated as fold change over *Optc* ^+/+^ and calculated as 2^ΔΔ^C_t_. Primer efficiencies for the genes under study were the same as those for the housekeeping gene, RPL19. The primer sequences were as described in Supplementary Material Fig. S2. Data were collected and processed with Rotor-Gene Q 6 software version 6.1 (Corbett Research, Mortlake, Australia). qPCR was performed in duplicate for each sample and gene studied.

### Sample collection and preparation

DMM and sham operated mice were euthanized at 10 weeks after surgery and the non-operated mice at 20 weeks old. Right knee joints were dissected free of tissue.

#### Histology

Samples were fixed in 4% paraformaldehyde, pH 7.4 for 16 hours at 4 °C (Sigma-Aldrich, Oakville, ON, Canada), decalcified in 10% EDTA pH 7.3 for 12 days (Wisent, St-Bruno, QC, Canada), and embedded in paraffin, as described^25^. Sections (5 µm) were deparaffinized in xylene followed by a graded series of alcohol washes prior to staining. Sections were stained with Safranin *O*–fast green (Sigma-Aldrich, Oakville).

The severity of the OA cartilage lesions was determined using the Osteoarthritis Research Society International (OARSI) scoring method^25^.

Subchondral bone plate histomorphometry was done on the medial compartment as described^23,26^ using a Leitz Diaplan microscope coupled to a personal computer as above using three Safranin-O/fast green stained sections. To measure the subchondral bone plate thickness, the bone volume/total volume ratio (%BV/TV) and trabecular thickness, a box with a fixed width (1,000 μm) and variable length was created with the upper limit at the calcified cartilage–subchondral bone junction and the lower limit at the subchondral bone–trabecular bone junction. The mean distance between the upper and lower limit was calculated automatically by the software as well as the trabecular thickness.

Histomorphometric quantitative analysis of the anterior synovial membrane thickness was performed by capturing images at 63X with a Leitz Diaplan microscope (Leica Microsystems, Wetzlar, Germany) coupled to a personal computer and data determined with Bioquant OSTEO Image Analysis Software version 12.5.60 MIR (Bioquant, Nashville, TN); data are expressed as micrometers.

#### Collagen organization

Cartilage collagen organization was evaluated on 5 µm paraffin sections following picrosirius red staining, as described^27^. In brief, each slide was evaluated under polarized light microscopy using a scale of 0–2, where 0 = no birefringence, completely disorganized collagen; 1 = birefringence on 25%-50% of the total cartilage thickness, collagen partially disorganized; and 2 = bright birefringence on more than 50% of total cartilage thickness, organized collagen network. Three areas for each slide were evaluated and the scores summed (maximum score 6).

#### Immunohistochemistry

Immunohistochemical analysis was performed on 5 µm paraffin sections as described^24^. The primary antibodies were against epiphycan, fibromodulin, lumican, opticin, C5b-9, CCL2, C terminal peptide of aggrecan G1 domain (VDIPEN), type II collagen primary cleavage site (Col2-3/4Cshort), type X collagen, MMP-3, and MMP-13. For further details see Supplementary Material, Materials and Methods. After incubation with an anti-rabbit IgG secondary antibody, slides were incubated with Vectastain ABC kit (Vector Laboratories, Burlingame, CA, USA) following the manufacturer’s instructions. The color was developed with 3,3′-diaminobenzidine containing hydrogen peroxide, and slides were counterstained with methyl green.

Control procedures were performed according to the same experimental protocol as follows: (1) omission of the primary antibody, (2) substitution of the primary antibody with a non-specific IgG from the same host as the primary antibody (Santa Cruz Biotechnology), and (3) a third control for type X collagen was performed by adsorption with the peptide YNRQQHYDPRSGIFTCKIPGIYYFSYGGC (provided by Dr. E. Lee, Shriners Hospital for Children, McGill University Hospital Centre, Montreal, Quebec, Canada) at 10-fold. Controls only showed background staining.

For each specimen, positive cells were quantified as previously described^23^. In brief, for each section, 1 microscopic field at 250X captured with a Leitz Diaplan microscope (Leica Microsystems) was assigned and chondrocytes staining positive were quantified following the determination of the total number of cells and of those staining positive for the antigen. Final results were expressed as the percentage of cells staining positive for the antigen with the maximum score being 100% for the entire cartilage, except for lumican which was expressed according to the upper (first half) or deep (second half) cartilage zone.

For type II collagen (Col2-3/4Cshort antibody) and VDIPEN, the matrix staining was assessed as follows. For type II collagen staining, images were captured at 250X with a Leitz Diaplan microscope connected to BIOQUANT OSTEO II Image Analysis software^23^. Surface area of positively stained extracellular cartilage matrix was measured and data expressed as % of positive stained area over total area. VDIPEN staining was graded on a scale of 0–3, where 0 = no staining; 1 = minor staining; 2 = marked staining; and 3 = maximal staining, as described previously^23^. Each slide was examined and scored by 2 independent observers who were blinded to group allocation.

### Transmission electron microscopy (TEM)

Right knees from 10-week-old *Optc* ^+/+^ and *Optc* ^−/−^ mice were used. For further details see Supplementary Material, Materials and Methods. Ultra-thin (100 nm) sections from the zone of interest were obtained using a microtome (Leica Microsystems UCT ultramicrotome, Vienna, Austria) and coated with 1–2 nm platinum. From each specimen, images were taken at magnification X1200 and X30000.

### Statistical analysis

Statistical significance was assessed by the Mann-Whitney and one sample t test where P values < 0.050 were considered significant.

### Data availability

The datasets generated and/or analysed during the current study are available from the corresponding author on reasonable request. Data generated or analysed during this study are included in this published article and its Supplementary Material files or are noted as data not shown.

## Results

### Confirmation of the lack of *Optc* expression in *Optc*^−/−^ mice

Confirmation of the lack of *Optc* expression in the *Optc* ^−/−^ mouse cartilage was performed using genomic DNA from 10-week-old mice. The signal observed at 350 bp in the samples obtained from *Optc* ^−/−^ mice confirmed the deletion in the region studied. Furthermore, expression of *Optc* mRNA extracted at day 3 (P03) from articular cartilage and processed for qPCR analysis as well as immunohistochemistry of 20-week-old DMM mice were done for the confirmation of OPTC protein deficiency in the cartilage. Results showed no signal in the chondrocytes or the extracellular matrix, suggesting the absence of OPTC (Supplementary Material Fig. S3).

### Mouse morphometric assessment

No significant differences were observed in growth (weight and length) between *Optc* ^−/−^ and *Optc* ^+/+^ mice as reported^21^ (Supplementary Material Fig. S4).

### Knee joint swelling

Data showed that the initial joint swelling (day 3 post-surgery) receded similarly in both *Optc* ^−/−^ and *Optc* ^*+/+*^ mice (Supplementary Material Fig. S5).

### *Optc*^−/−^ mice exhibited a protective effect on cartilage degradation

**Figure 1:**
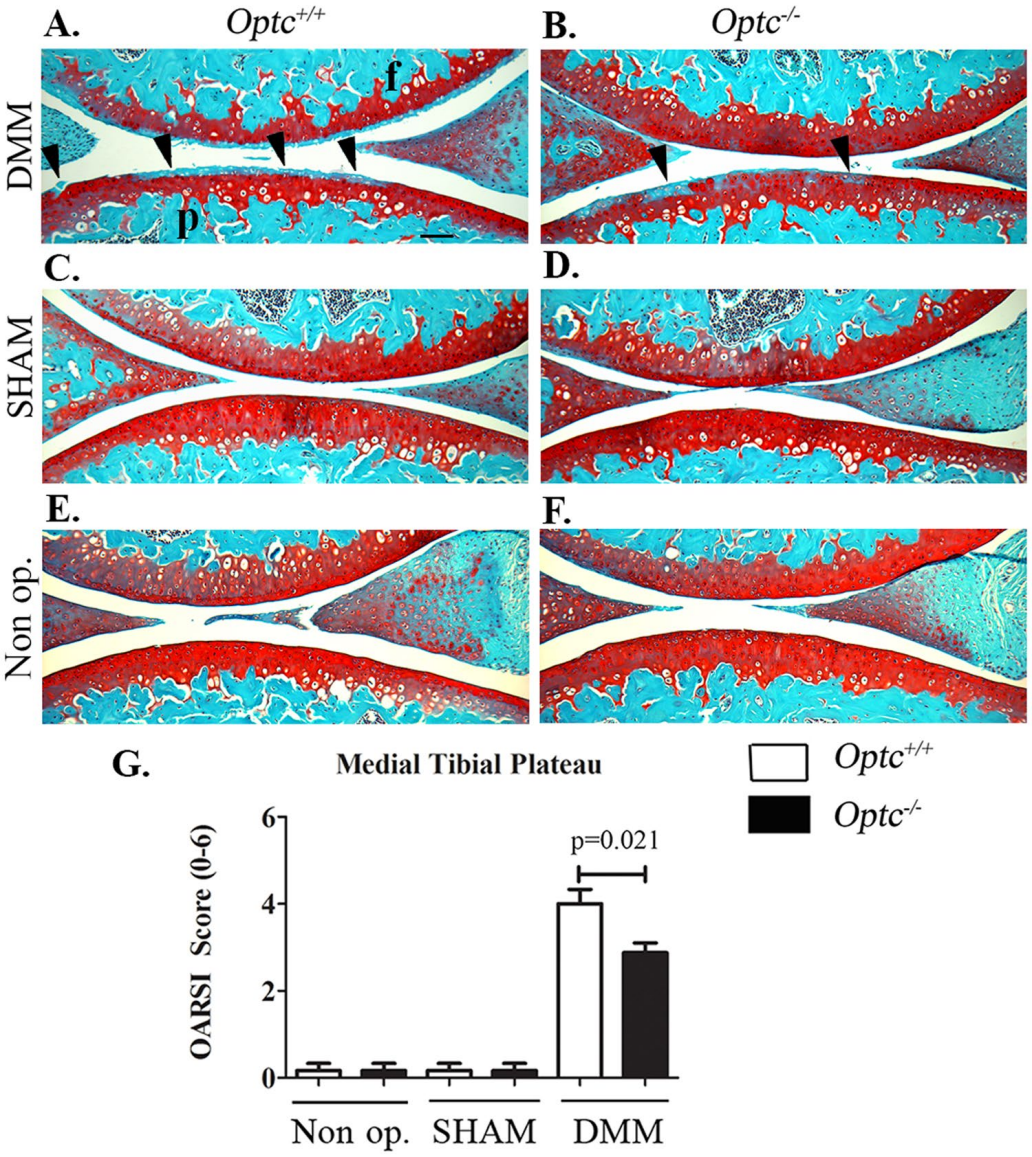
Histology of articular cartilage. Photomicrographs of representative histological sections of the (**A**,p) medial tibial plateaus and (**A**,f) femoral condyles obtained from (**A**,**C**,**E**) opticin *Optc* ^+/+^ and (**B**,**D**,**F**) *Optc* ^−/−^ mice at 10 weeks after (**A**,**B**) DMM, (**C**,**D**) sham (control) surgery, and (**E**,**F**) non-operated (Non op.; control). Black arrowheads in (**A** and **B**) indicate areas of fibrillation, with loss of cartilage ultrastructure. Bar in (**A**) = 100 μm. Original magnification X100. (**G**) Representative histograms of the Osteoarthritis Research Society International (OARSI) scores in the medial tibial plateau. Values are the mean ± SEM of Non op. (*Optc* ^+/+^, n = 4; *Optc* ^−/−^, n = 7), sham (n = 6/group), DMM-*Optc* ^+/+^ (n = 9) and DMM-*Optc* ^−/−^ (n = 8). p values were determined by Mann-Whitney test; only significant p values are shown except for those between Non op./sham with respective DMM groups in medial tibial plateau which are < 0.010.

The cartilage integrity in the medial tibial plateau in non-operated, sham and DMM (OA) mice was assessed histologically (Fig. 1). No significant differences among the control groups (20-week-old non-operated and sham) of *Optc* ^−/−^ and *Optc* ^+/+^ mice were found (Fig. 1). Comparison between DMM*-Optc* ^+/+^ and -*Optc* ^−/−^ mice (Fig. 1A,B) revealed a decreased loss of cartilage integrity in the *Optc* ^−/−^ mice, including Safranin *O*–fast green staining, cellularity, thinning of the cartilage, and fibrillation. These observations were corroborated by the OARSI scores (Fig. 1G), which showed significantly lower histologic scores in the DMM*-Optc* ^−/−^ mice (p = 0.021).

### Less cartilage degradative markers in DMM*-Optc*^−/−^

**Figure 2:**
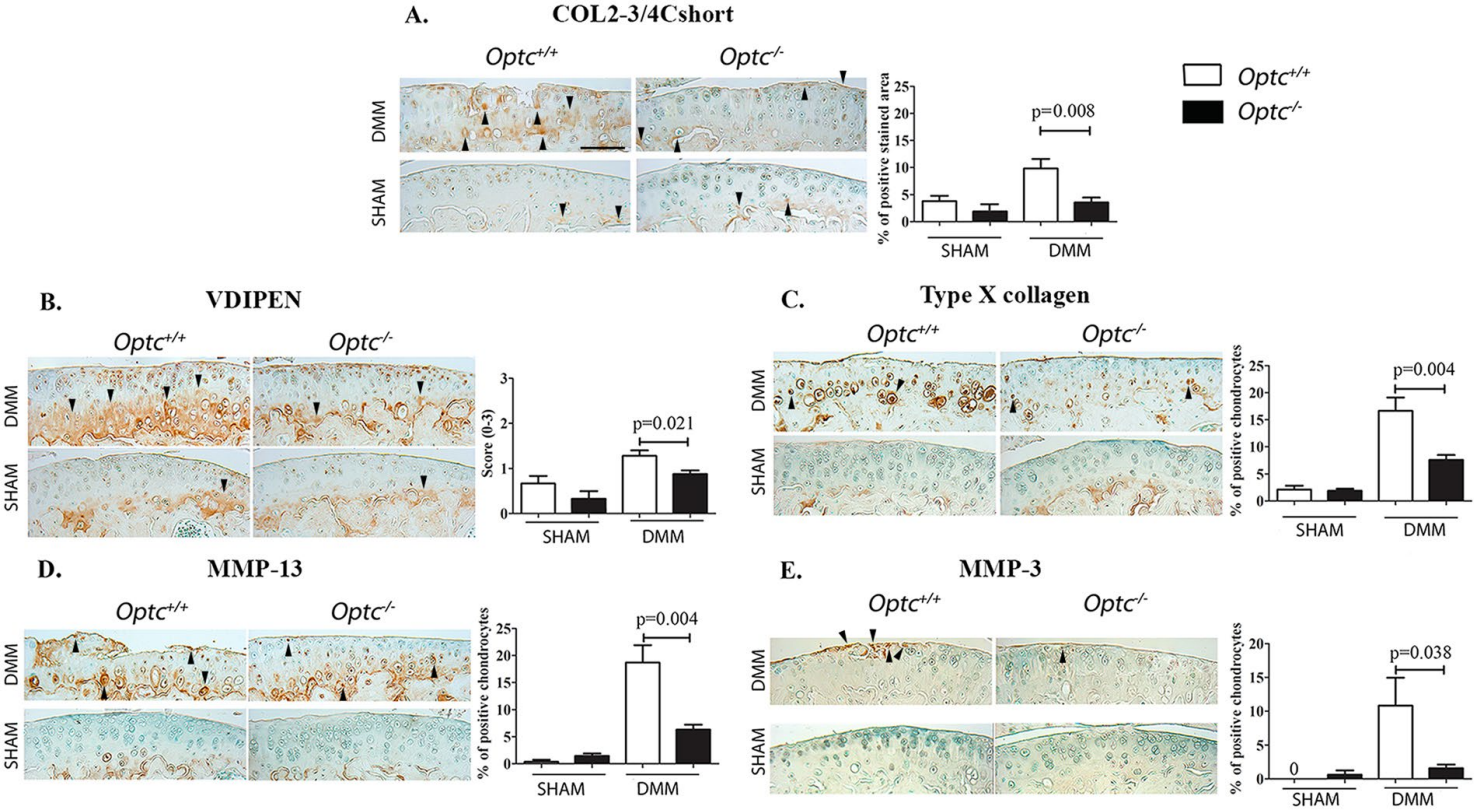
Immunohistochemistry of articular cartilage 10 weeks after DMM surgery. Representative sections and histograms of the percentage of positive chondrocytes or matrix staining of cartilage degradation markers in medial tibial plateaus (**A**–**E**) from *Optc* ^+/+^ and *Optc* ^−/−^ mice at 10 weeks after sham (control) and DMM surgery, counterstained with methyl green. Black arrowheads indicate stained area (**A**,**B**) or positive-stained cells (**C**,**D**,**E**). Bar in (**A**) = 100 μm. Original magnification X250. Values are the mean ± SEM of sham (control) (n = 6/group), DMM-*Optc* ^+/+^ (n = 9) and DMM-*Optc* ^−/−^ (n = 8). p values were determined by Mann-Whitney test; only significant p values are shown except for those between sham and their respective DMM groups which are < 0.050. Negative controls are shown in Supplementary Material Fig. S10.

According to the histology, DMM*-Optc* ^−/−^ compared to -*Optc* ^+/+^ mice demonstrated significantly reduced levels of α1 chain of type II collagen (Col2-3/4Cshort; p = 0.008) (Fig. 2A); aggrecan degradation products (VDIPEN; p = 0.021) (Fig. 2B); type X collagen (p = 0.004) (Fig. 2C), MMP-13 (p = 0.004) (Fig. 2D) and MMP-3 (p = 0.038) (Fig. 2E).

### *Optc*^−/−^ present different SLRP expression/production in articular cartilage

**Figure 3:**
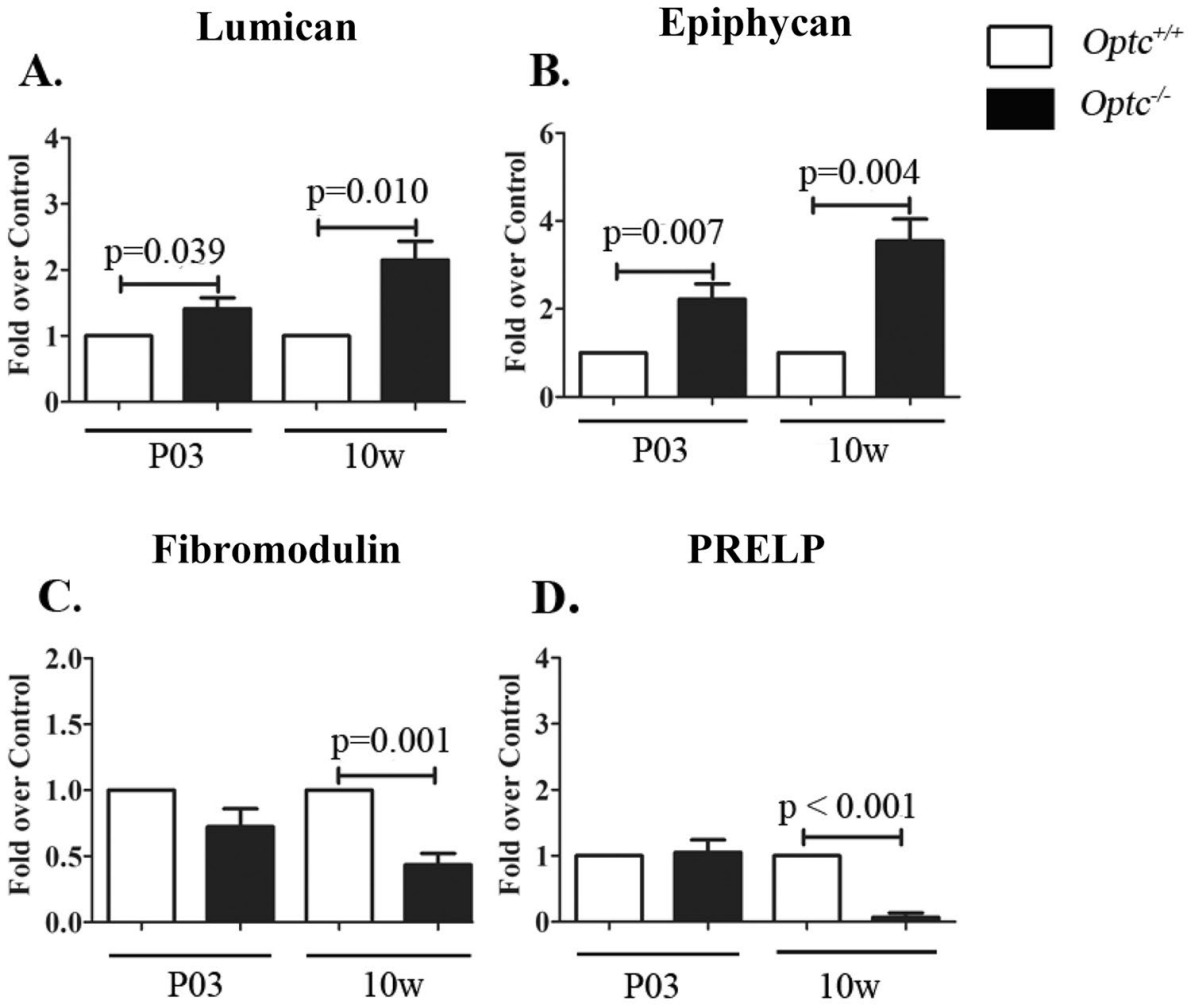
Expression of small leucine-rich proteoglycans (SLRPs) in articular cartilage. Relative gene expression (mRNA) (**A**–**D**) in articular cartilage in day 3 (P03) and 10-week-old (10 w) *Optc* ^+/+^ and *Optc* ^−/−^ mice. Values are the mean ± SEM of (n = 6/group) mice. p values were determined by one-sample t test with *Optc* ^+/+^ value of 1.

Analysis of the expression (mRNA) of SLRP members from each class revealed that biglycan (class I), osteomodulin (class II), osteoglycin (class III), tsukushi and nyctalopin (class IV), and podocan (class V) expression showed no significant difference between non-operated *Optc* ^+/+^ and *Optc* ^−/−^ mice at either day 3 (P03) or at 10 weeks of age in articular cartilage (data not shown). Interestingly, lumican (class II) (Fig. 3A) and epiphycan (Fig. 3B), a member of the same class as OPTC (class III), were significantly overexpressed at both P03 (p ≤ 0.039) and at 10 weeks of age (p ≤ 0.010) in *Optc* ^−/−^ compared to *Optc* ^+/+^ mice. Moreover, compared to *Optc* ^+/+^, in *Optc* ^−/−^ mice fibromodulin (class II) (Fig. 3C) and PRELP (class II) (Fig. 3D) had similar values at P03, but were significantly down-regulated at 10 weeks of age (p < 0.001) (Fig. 3C,D).

**Figure 4:**
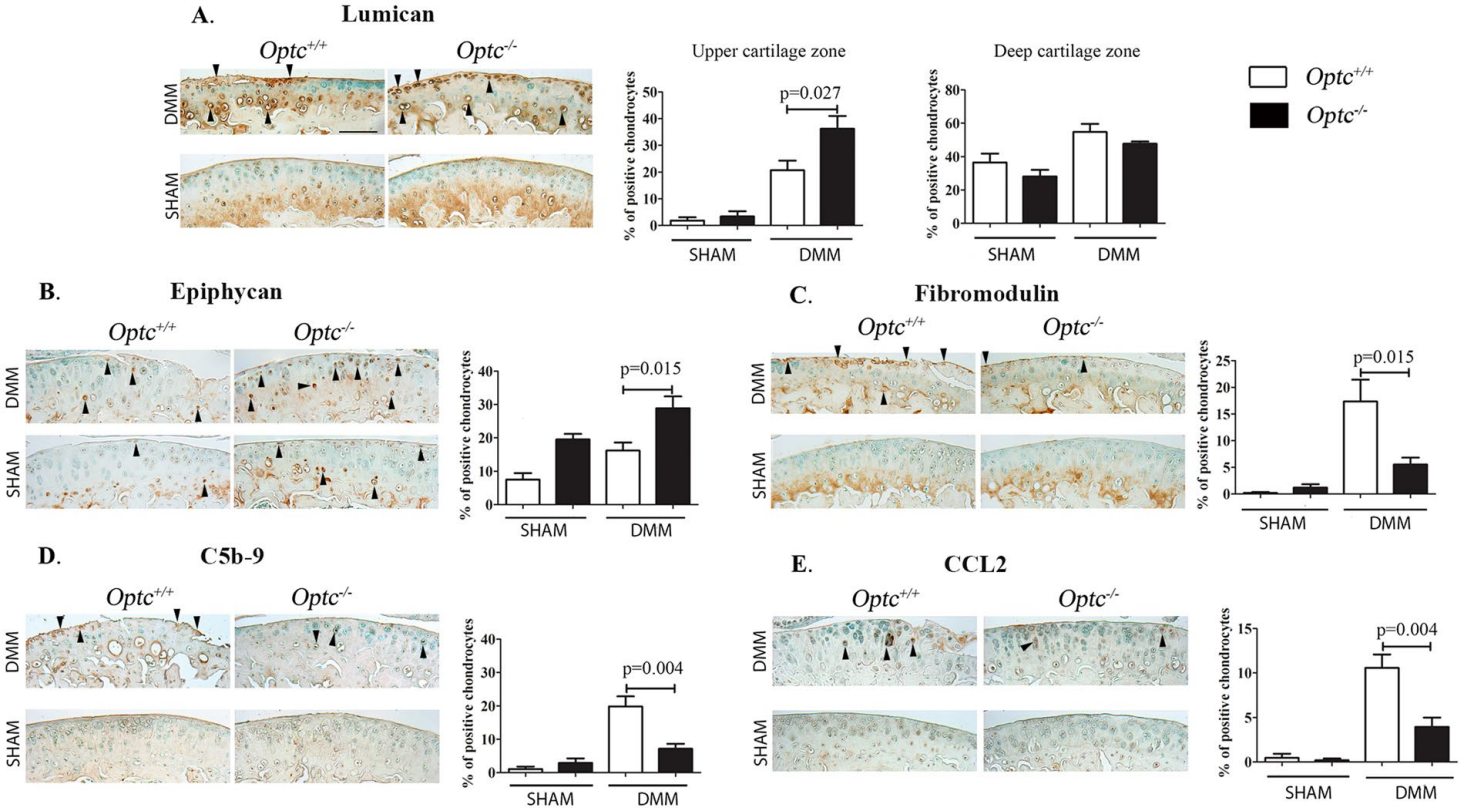
Immunohistochemistry of articular cartilage 10 weeks after DMM surgery. Representative images and histograms of the percentage of positive chondrocytes of SLRPs (**A**–**E**) from *Optc* ^+/+^ and *Optc* ^−/−^ mice at 10 weeks after sham (control) and DMM surgery, counterstained with methyl green. Controls performed with non-specific IgG and in addition for type X collagen, by adsorption with the peptide are shown in Supplementary Material Fig. S10. Black arrows indicate positive-stained cells. Bar in (**A**) = 100 μm. Original magnification X250. Values are the mean ± SEM of sham (n = 6/group), DMM-*Optc* ^+/+^ (n = 9) and DMM-*Optc* ^−/−^ (n = 8). p values were determined by Mann-Whitney test; only significant p values are shown except for those between sham and their respective DMM groups which are < 0.010 except for epiphycan and C5b-9 between sham and DMM-*Optc* ^−/−^. Negative controls are shown in Supplementary Material Fig. S10.

Complementary analysis comparing cartilage immunohistochemistry of DMM-*Optc* ^+/+^ and DMM-*Optc* ^−/−^ mice showed significantly increased levels in chondrocyte staining of lumican in the upper cartilage zone (p = 0.027) (Fig. 4A) and epiphycan (p = 0.015) (Fig. 4B), but a significant decrease for fibromodulin (p = 0.015) (Fig. 4C), concurring with the expression findings (Fig. 3). On the other hand, PRELP levels did not show differences between DMM-*Optc* ^−/−^ and -*Optc* ^+/+^ mouse cartilage (data not shown).

As fibromodulin contributes to an increase in the complement activation/inflammatory process, we further looked at the levels of two factors of the complement activation, C5b-9 and CCL2. Data revealed that for both factors, DMM*-Optc* ^−/−^ mice demonstrated a significantly decreased level of positive chondrocytes (p = 0.004) compared to -*Optc* ^+/+^ mice (Fig. 4C,D).

### Collagen fibers of *Optc*^−/−^ mice were thinner and better organized than *Optc*^*+/+*^mice

**Figure 5:**
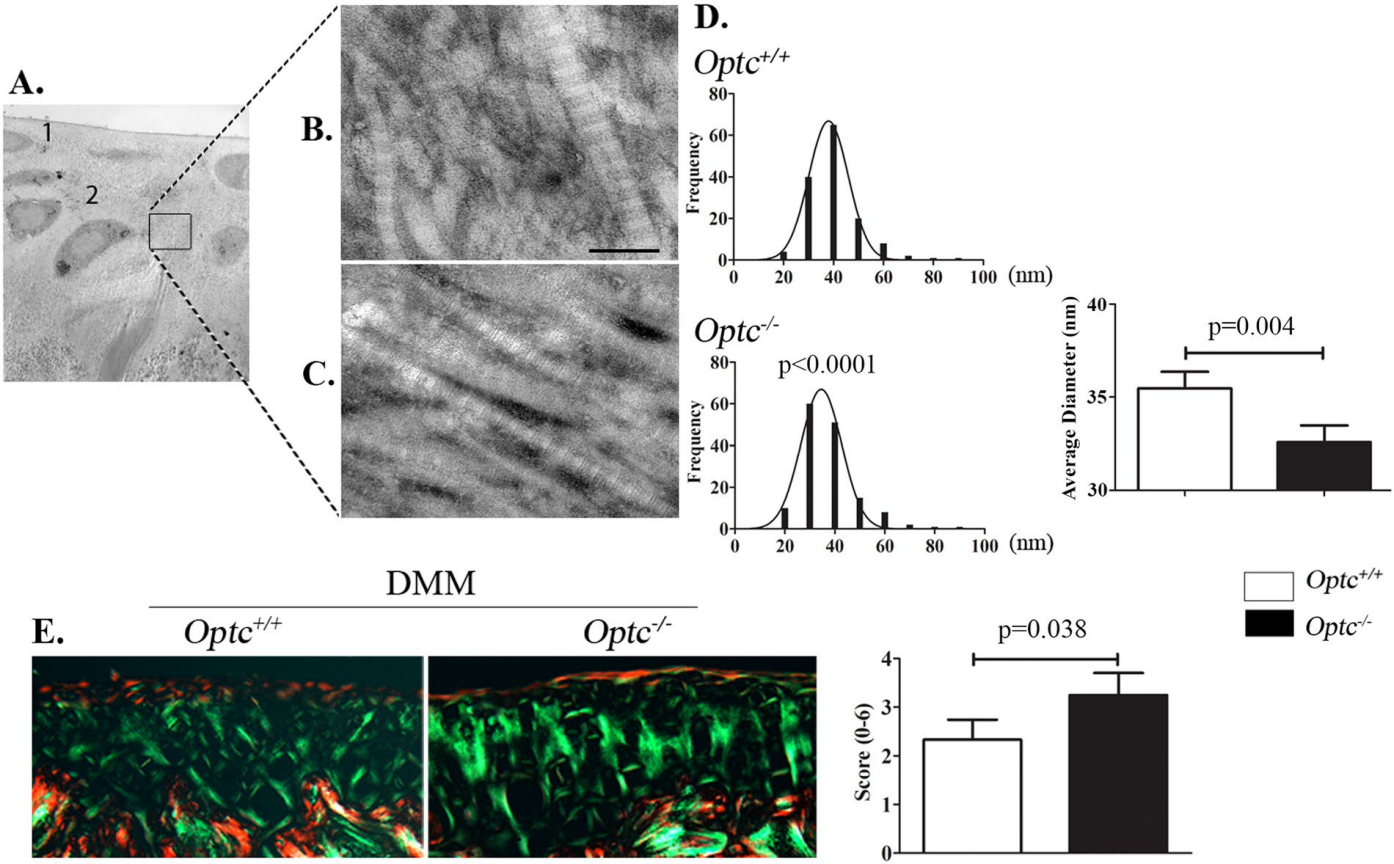
Ultrastructure of type II collagen fibers. Representative images obtained with transmission electron microscopy (TEM) of (**A**) cartilage, in which 1 indicates the upper zone and 2 the deep zone, and collagen fibers from (**B**) *Optc* ^+/+^ and (**C**) *Optc* ^−/−^ mice at 10 weeks of age. Bar in (**B**) = 100 μm. Magnifications: (**A**) X1200 and (**B**,**C**) X30000. (**D**) Histograms represent the frequency of each fiber’s diameter and the average diameter of *Optc* ^+/+^ and *Optc* ^−/−^ mice. (**E**) Picrosirius red staining of mouse cartilage from DMM-*Optc* ^+/+^ and DMM-*Optc* ^−/−^ mice and the histogram representing the birefringence score. Values in graphs in (**D**,**E**) are mean ± SEM. p values were determined by Mann-Whitney test.

Fibril diameter distributions were analyzed by TEM in the upper and deep zones of the cartilage (Fig. 5A–C). Data showed similar findings for both zones studied and only data of the deep zone are illustrated (Fig. 5B,C). Findings revealed that *Optc* ^−/−^ mice had a significantly higher frequency of cartilage collagen fibers of smaller diameter compared to *Optc* ^+/+^ mice (Fig. 5D). TEM images also showed that in *Optc* ^−/−^ mice, type II collagen fibers were more tightly packed than in *Optc* ^+/+^ mice. This was further substantiated by the use of the collagen birefringence methodology (Fig. 5E), in which collagen fibers in the DMM-*Optc* ^+/+^ mouse cartilage had a significantly lower birefringence intensity (p = 0.038) than in the -*Optc* ^−/−^ mice, indicating a higher disorganization of the collagen fibers in the *Optc* ^+/+^ mice.

### *Optc*^−/−^ effect on two other articular tissues

As OPTC was also found present in both osteoblasts and synoviocytes^19^, we further looked at the effect of *Optc* deficiency in both subchondral bone and synovial membrane. Histomophometric evaluations of the subchondral bone: plate thickness, percentage of bone volume/total volume and trabecular thickness, demonstrated similar values for the DMM-*Optc* ^+/+^ and -*Optc* ^−/−^ mice (Supplementary Material Fig. S6). However, synovial membrane thickness comparison of DMM-*Optc* ^+/+^ with -*Optc* ^−/−^ revealed a significant increase (p = 0.036) (Supplementary Material Fig. S7).

## Discussion

Our group previously reported that OPTC is expressed and produced in articular cartilage and degraded during human OA^19,20^. Because SLRPs contribute to the maintenance and integrity of articular cartilage, OPTC reduction/cleavage in OA was suggested to predispose cartilage to degeneration. Here, we further investigated the *in vivo* effect of the lack of OPTC during OA, using an *Optc*-null mouse model.

Although mice with a deficiency of one or more SLRPs have been shown to develop premature OA^11,13^, unexpectedly and in contrast to our hypothesis, the present study showed that OA *Optc*-null mice exhibited a cartilage protective effect. The lack of OPTC in OA mice resulted in significantly better cartilage histology features and lower expression markers of catabolic processes. We then further explored the relevant *in vivo* mechanisms implicated in OA cartilage associated with the OPTC absence. Data revealed that this ensues from an overexpression of two other members of the SRLP super-family, lumican and epiphycan, combined with a down-regulation of fibromodulin, a factor involved in activation of the complement/inflammatory process. These changes are associated with a better preservation of the collagen fibril organization observed in the *Optc*-null mice.

Our observation of the cartilage protection in OA *Optc* ^−/−^ mice, together with the literature demonstrating that the lack of some members of the SLRP super-family is compensated by an overexpression of other members^12,16,28,29^, led us to investigate the expression of SLRPs from each class on articular mouse cartilage at birth and in adults. Data revealed that in *Optc* ^−/−^ mice, lumican and epiphycan are overexpressed at birth (P03) and in adults (10 weeks old), and fibromodulin down-regulated in adult mice. Such overexpression of lumican in adult mice has also been reported in the tendons when fibromodulin is absent^15^, and agrees with our data on the significant decrease in fibromodulin in adult *Optc* ^−/−^ mice. The epiphycan overexpression (SLRP belonging to the same class as OPTC) also concurs with cartilage protection during OA, as this SLRP was shown to play an important role in maintaining joint integrity and its absence (knockout mice) resulted in OA development with aging^13^. In addition, interaction between SLRP members has also been documented; for example, epiphycan/biglycan double-deficient mice present a more severe OA than only epiphycan knock-out mice^13^.

Findings of the OA *Optc* ^−/−^ cartilage protective effect also concur with those of the markers of the cartilage degradation, including products of type II collagen and aggrecan, as well as chondrocyte hypertrophy, MMP-3 and MMP-13, which were all significantly and markedly reduced in the DMM*-Optc* ^−/−^ compared to DMM-*Optc* ^*+/+*^ mice.

SLRPs are encoded in 18 different genes clustered on 7 chromosomes, suggesting duplication to generate functional redundancy during evolution^2^. Interestingly, OPTC and fibromodulin share the same gene cluster, suggesting that OPTC could have a regulatory role in fibromodulin transcription^18^. This could explain, at least in part, the decrease in fibromodulin expression in *Optc* ^−/−^ mouse cartilage. In addition, it could be that fibromodulin degradation by proteases is high in OA cartilage or at least higher than other SLRPs. Indeed, previous data reported that although MMP-13, a major MMP involved in collagen degradation, is able to cleave both lumican and fibromodulin, fibromodulin appears to be the preferred substrate^30^.

Collagen fibrillogenesis during normal cartilage development is finely regulated and involves multiple steps. Type II collagen fibrils impart a strength and compressibility to the cartilage matrix and permit this tissue to resist large deformations in shape, allowing joints to absorb shocks. During the first steps of fibrillogenesis, a population of collagen molecules is assembled into quarter staggered arrays forming short small-diameter fibril intermediates^31^ (Supplementary Material Fig. S8). After the molecular assembly phase, fibrils start growing and become longer and larger in diameter and the stabilization of the assembled fibrils is mediated by interactions with fibril-associated molecules such as SLRPs^29^, more specifically by lumican and fibromodulin, two SLRPs differently regulated in the *Optc* ^−/−^ mice. Lumican and fibromodulin belong to the same SLRP subfamily (class II) and show a 47% sequence identity. Additionally, these SLRPs are similar in their post-translational modifications. Both carry tyrosine sulfate residues in their N-terminal domains and are substituted with carbohydrates in a similar manner^32^. They bind to the same binding site of type I collagen fibrils, although fibromodulin has a higher affinity than lumican^33^. Even though their binding characterization to type II collagen has not been studied, one could speculate that these two SLRPs could also compete for binding in this collagen type. Moreover, data from tendon development studies in lumican-, fibromodulin-, and double-deficient mice suggest a collagen fibrillogenesis model where fibromodulin would displace lumican at the fibril maturation stage^29^ (Supplementary Material Fig. S8). At the beginning of the collagen fibril growth phase, lumican promotes the lateral fibril growth by fusion of intermediate subunits and, as fibrillogenesis progresses, the lumican level decreases to a barely detectable level, while the fibromodulin level increases significantly and is essential for diameter growth of the mature fiber at later stages^29^.

These data, together with our findings on the SLRP expression/production and the cartilage collagen analysis, and further experiments in which the *Optc* ^+/+^ lumican, epiphycan and fibromodulin fold change expression (mRNA) in 10-week-old mice over P03 mice was investigated and found down-regulated at 10 weeks old (Supplementary Material Fig. S9), are suggestive of a collagen fibrillogenesis model in the absence of OPTC (Supplementary Material Fig. S8).The decreased level of fibromodulin is translated by a compensatory overexpression of lumican over time. As a result, the collagen fibers of the *Optc* ^−/−^ mice are over-coated by lumican and epiphycan molecules during the fibrillogenesis process, preventing the final steps of the fibril process from developing and the fibers to fuse laterally, thus remaining thinner and tightly packed, and therefore less susceptible to degradation. This scheme is strengthened by the picrosirius red data that showed that OA-*Optc* ^−/−^ mice maintained a better organization of the cartilage collagen network than OA-*Optc* ^+/+^ mice.

In turn, the decrease in fibromodulin in *Optc* ^−/−^ mice could be responsible for less catabolic activity in the cartilage. Indeed, fibromodulin, in addition to its structural role in the cartilage matrix, is known to activate the classical complement pathway^9^. Unbalanced complement activation has been shown to play a central role in OA cartilage^34^. The complement system consists of pathways that converge at the formation of the C3 and C5 convertases, enzymes that mediate activation, among others, of the formation of membrane attack complex (MAC), comprising the complement effector C5b-9^35^. The levels of C5b-9 have been found elevated in OA synovial fluid when compared to healthy individuals^34,36^. Interestingly, MAC co-localized with MMP-13 around human OA chondrocytes and was shown to play a critical role in the pathophysiology of this disease, in which in addition to inducing cell lysis, it increases the chondrocytes’ expression of multiple genes including MMPs, inflammatory cytokines and complement effectors^34^. We then looked at the effect of the lack of OPTC on two representative complement factors, C5b-9 and CCL2 (also referred to as monocyte chemoattractant protein 1 [MCP-1]), which have been found significantly increased in serum and synovial fluid of human OA patients^34,36,37^. Data revealed that the levels of these two factors were significantly decreased in OA-*Optc* ^−/−^ compared to -*Optc* ^+/+^ mice contributing to a reduction in the inflammatory/catabolic processes during the disease process.

Although the hallmark of OA is the progressive degeneration of articular cartilage, it is acknowledged that the subchondral bone and synovial membrane alterations are also components of the disease process. An interdependence between subchondral bone alterations and cartilage degeneration, as well as inflammatory factors released from the synovial membrane, is gaining strong support. As data showed that OPTC deficiency imparts a protective effect on the synovial membrane thickness, but not on the subchondral bone, the question arises as to whether a protective effect on the synovial membrane alterations could also be beneficial to the cartilage in this OA mouse model. Although more research should be performed to that effect, in this OA model it is noteworthy that inflammation of the synovial membrane is not the main feature^38^ and that OA alterations in this tissue are more related to non-inflammatory progressive fibrosis. However, it could be that the synovial membrane preservation occurs via a direct effect of the cartilage preservation, as less degradation products will be released from the cartilage.

In summary, this is the first report showing that the *in vivo* lack of OPTC destabilizes the natural balance of SLRP members in the cartilage, which is translated into a protective environment in OA cartilage. Such effect is a result of different events comprising an overexpression of lumican and epiphycan expression/production at birth and over time and therefore a protection of the collagen fibers by their surface deposition, enabling the fibrils to better resist catabolic factors. In addition, the protective effect also reflects the decrease in fibromodulin, which will result in a reduction of the classical complement pathway activation during the progression of the disease, favoring production of less catabolic factors, thus less degradation.

In conclusion, although OPTC, a SLRP associated with collagen fibrils, contributes to the structural stability of cartilage, its lack leads to a protection of cartilage due to an overexpression of other SLRP members throughout adult life, thus preserving the collagen fibril from degradation, and by a reduction in the complement activation/inflammatory process in this tissue during OA through the reduction of another member. This work suggests that the evaluation of the composition of the different SLRPs in human OA cartilage could be applied as a new tool for OA prognosis classification.

## Electronic supplementary material

Supplementary Material
