# Supplementary material for: In vivo effect of opticin deficiency in cartilage in a surgically induced mouse model of osteoarthritis
Source: Sci Rep. 2018 Jan 11;8:457. doi: 10.1038/s41598-017-18047-w (PMC5765138; doi:10.1038/s41598-017-18047-w)
Supplement: Supplementary file 1 — Supplementary Material [file 41598_2017_18047_MOESM1_ESM.pdf]

In vivo effect of opticin deficiency in cartilage in a surgically induced mouse model of osteoarthritis

Aina Farrán, Gladys Valverde-Franco, Laura Tío, Bertrand Lussier, Hassan Fahmi, Jean-Pierre Pelletier, Paul N. Bishop, Jordi Monfort, Johanne Martel-Pelletier

SUPPLEMENTARY MATERIAL

MATERIALS AND METHODS

Mice

Frozen heterozygous embryos (*Optc*$^{+/-}$) were provided by Dr. Bishop, University of Manchester, UK [1]. Briefly, frozen embryos were thawed and transferred to recipient 'pseudo-pregnant' albino CD-1 (Charles River Laboratories, Shrewsbury, MA, USA) females. The *Optc*$^{+/-}$ mice became our foundation stock. They were bred until having pure *Optc*$^{-/-}$ (knock-out) and *Optc*$^{+/+}$ (wild type). Routine genotyping was carried out on DNA extracted from ear punch biopsy samples using specific primers. Genomic DNA was isolated with Direct PCR lysis reagent containing proteinase K (Qiagen Biotech, Valencia, CA, USA) according to the manufacturer's instructions. The polymerase chain reaction (PCR) was performed on genomic DNA using the following primers: 5'-CAAGCTCCATCCATCCAAGAA-3' and 5'-TGTGGCTGATGTGGTTGAA-3' for *Optc*$^{-/-}$; 5'-CAAGCTCCATCCATCCAAGAA-3' and 5'-CTTCTGGTGGAGATGATGACTG-3' for *Optc*$^{+/+}$, that generate fragments of 450bp and 350bp, respectively. Schematic representation of the *Optc*$^{-/-}$ construct and primer design is illustrated in Supplementary Material, Fig. S1 below.

Surgically induced OA mouse model

In brief, mice were anesthetized with isoflurane and O_2, and an arthrotomy of the right knee joint was performed and the medial meniscus destabilized by transection of the anterior menisco-tibial ligament (AMTL). A sham surgery, which involves only an arthrotomy without compromising the AMTL, was also performed on the right knee of the 10-week-old *Optc*$^{+/+}$ and *Optc*$^{-/-}$ mice as controls. Mice had free access to an exercise wheel after the surgery, and were observed daily to verify healing and to ensure the use of right limbs.

Sample collection and preparation

Immunohistochemistry

Immunohistochemical analysis was performed on 5 µm paraffin sections which were sequentially pretreated with 0.25 units/ml of protease-free chondroitinase ABC in 0.1 M tris-acetate buffer for 60 minutes at 37°C (Sigma-Aldrich), with 1% hyaluronidase pH 6.0 in PBS (Sigma-Aldrich) for 60 minutes at 37°C (only for VDIPEN, Col2-3/4short and opticin antibodies), with 0.1% collagenase pH 7.4, $CaCl_2$ 0.1% for 60 minutes at 37°C (for epiphycan, MMP-3 and C5b-9 antibodies), with 0.01M citrate buffer for 20 minutes at 68°C (for epiphycan, CCL2 and opticin antibodies), and at room temperature with 0.3% triton X-100 for 30 minutes, with hydrogen peroxide 2% for 15 minutes and then with normal goat serum 2% for 45 minutes. The specimens were incubated for 18 hours at 4ºC with the following primary antibodies: rabbit polyclonal anti-human against epiphycan (1:50 dilution; Sigma-Aldrich), rabbit polyclonal anti-

human against fibromodulin (1:100 dilution; ThermoFisher, Waltham, MA, USA), rabbit polyclonal anti-mouse against lumican (1:50 dilution; ThermoFisher), rabbit polyclonal anti-human against opticin (1:25 dilution; Novus Biologicals, Oakville, ON, Canada), rabbit polyclonal anti-human against C5b-9 (1:500 dilution; Abcam, Toronto, ON, Canada), rabbit polyclonal anti-rat against CCL2 (1:200 dilution; Abcam), rabbit polyclonal anti-C terminal peptide of aggrecan G1 domain (VDIPEN, 1:800 dilution; provided by Dr. J. S. Mort, Shriners Hospital for Children, McGill University Hospital Centre, Montreal, Quebec, Canada) [2], rabbit polyclonal antibody that represents a type II collagen primary cleavage site (Col2-3/4Cshort, 1:250 dilution; Ibex, Montreal, QC, Canada), rabbit polyclonal anti-rat type X collagen antibody (1:2000 dilution; Abcam), rabbit monoclonal anti-human against MMP-3 (1:75 dilution; Abcam) and rabbit polyclonal anti-human against MMP-13 (1:100 dilution; Sigma-Aldrich).

Transmission electron microscopy (TEM)

Knees from OPTC mice were dissected and immediately fixed in 2.5% glutaraldehyde in 0.1 M cacodylate buffer for 12 hours at 4ºC, 1% glutaraldehyde in double distilled water for 10 days and post-fixed with 1% aqueous osmium tetroxide and 1.5% aqueous potassium ferrocyanide for 2 hours at 4ºC. Samples were dehydrated though a series of increasing concentrations of acetone: 30%, 50%, 70%, 80%, 3X100%, each for 8 minutes, and infiltrated with epon epoxy resin (Cedarlane, Burlington, ON, Canada)/acetone at increasing ratios 1:1, 2:1 and 3:1 during 48 hours in a rotator. The tissues were embedded in a glass mold containing epon epoxy resin and polymerized at 60ºC during 48 hours.

1 Le Goff MM, Lu H, Ugarte M, et al. The vitreous glycoprotein opticin inhibits preretinal neovascularization. Investigative Ophthalmology and Visual Science 2012;53(1):228-34.

2 Hughes CE, Caterson B, Fosang AJ, Roughley PJ, Mort JS. Monoclonal antibodies that specifically recognize neoepitope sequences generated by 'aggrecanase' and matrix metalloproteinase cleavage of aggrecan: application to catabolism in situ and in vitro. Biochemical Journal 1995;305(Pt 3):799-804.

A.

B.

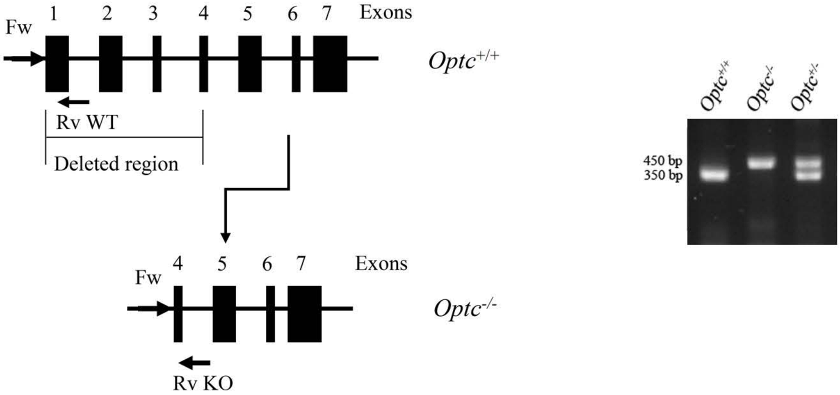

Supplementary Fig. S1. Genotyping of *Optc mice.* **(A)** Representative diagram of mouse *Optc* gene indicating region deleted in *Optc*$^{-/-}$ mice and position of genotyping primers forward (Fw), reverse wild-type (Rv WT) and reverse knockout (Rv KO). **(B)** Electrophoresis separation of genotyping PCR products differentiating *Optc* knockout (*Optc*$^{-/-}$), wild-type (*Optc*$^{+/+}$), and heterozygous (*Optc*$^{+/-}$) with their respective sizes in base pairs (bp). Full-length gel is shown in Supplementary Fig. S11.

Gene	Sense	Anti-sense
Asporin	5’-GGTCAGGGGCAAATACCAAGGACTCT-3’	5‘-CTCTACATGGTTGTCAGGAATGTG-3’
Biglycan	5’-CTGAGGGAACTTCACTTGGA-3’	5‘-CAGATAGACAACCTGGAGGAG-3’
Chondroadherin	5’-CAGTCTGGTCTTTCTTGCCA-3’	5’-ATGTCGTTGTGGGACAGGTA-3’
Decorin	5’-TTGTCATAGAACTGGGCGGC-3’	5’-CAGACCTTGAGGGATCGCAG-3’
Epiphycan	5’-GGTCAGGGGCAAATACCAAGGACTTCT-3’	5’-CTCTACATGGTTGTCAGGAATGTG-3’
Fibromodulin	5’-TCAACCCAAGAGACAAAAATGCAG-3’	5’-CTCAGAGGGCTCATAGGGGT-3’
Lumican	5’-GAGTAAGGTCACAGAGGACTTGC-3’	5’-ATTCTGGTGCACAGTTGGGT-3’
Nyctalopyn	5’-GCCGGGTTTTAAAGCATACA-3’	5’-GCCTGACACCCAAAGTTGTT-3’
Opticin	5’-GGGATTCAGCCATTCCCACT-3’	5’-CCTCAGGGCTCTGCTCTTCT-3’
Osteoglycin	5’-CTACTGTGAAGAAGTTGACATTGATGCTG-3’	5’-GGTAAATTAGGAGGCACAGATTCCAGG-3’
Podocan	5’-GCAGGAGGATGAGCATTAGC-3’	5’-TCTGGTCATTTGGGCTTTTC-3’
PRELP	5’-CTGCAGTCCGTGGTCATCTA-3’	5’-TTTAGAGCCGGAGCAGGTTA
Tsukushi	5’-GAACCCTCTGGCTACCATCA-3’	5’-GCCTGAAAACACCTCAGCTC-3’

Supplementary Fig. S2. Sequences of the mouse gene-specific primers used for qPCR

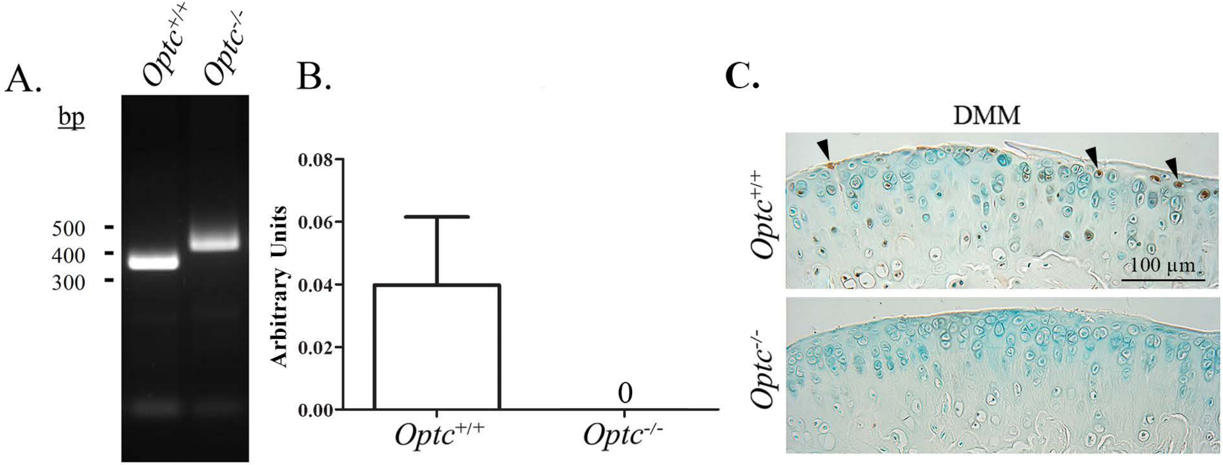

Supplementary Fig. S3. OPTC expression and production in mouse cartilage. (A) Genomic DNA was extracted from articular cartilage from both *Optc*$^{+/+}$ and *Optc*$^{-/-}$ mice and processed for PCR using primers that differentiated between *Optc*$^{+/+}$ (350 bp) and *Optc*$^{-/-}$ (450 bp) mice, respectively. Full-length gel is shown in Supplementary Fig. S12. **(B)** Expression of OPTC mRNA extracted from articular cartilage and processed for qPCR analysis. *Optc*$^{+/+}$ and *Optc*$^{-/-}$ (n=4 each group) values were calculated as the ratio of the number of molecules of OPTC/the number of molecules of the housekeeping gene RPL19 and data expressed as mean SEM of arbitrary units. **(C)** Immunohistochemistry of OPTC on articular cartilage of *Optc*$^{+/+}$ and *Optc*$^{-/-}$ mice at 10 weeks after DMM surgery. Arrowheads in **(C)** indicate positive stained cells. Original magnification X250.

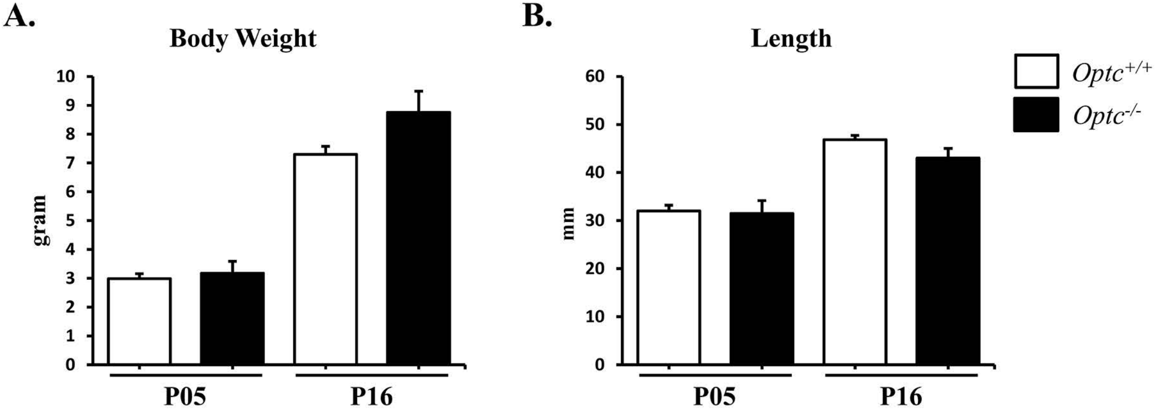

Supplementary Fig. S4. Morphometric assessments. Histograms show **(A)** body weight and **(B)** length of *Optc*$^{+/+}$ and *Optc*$^{-/-}$ mice at day 5 (P05)(n=6 and n=4, respectively) and P16 (n=6 and n=4). Values are mean±SEM. p values were determined by Mann-Whitney test and there was no statistically significant difference between each *Optc*$^{+/+}$ and *Optc*$^{-/-}$ group.

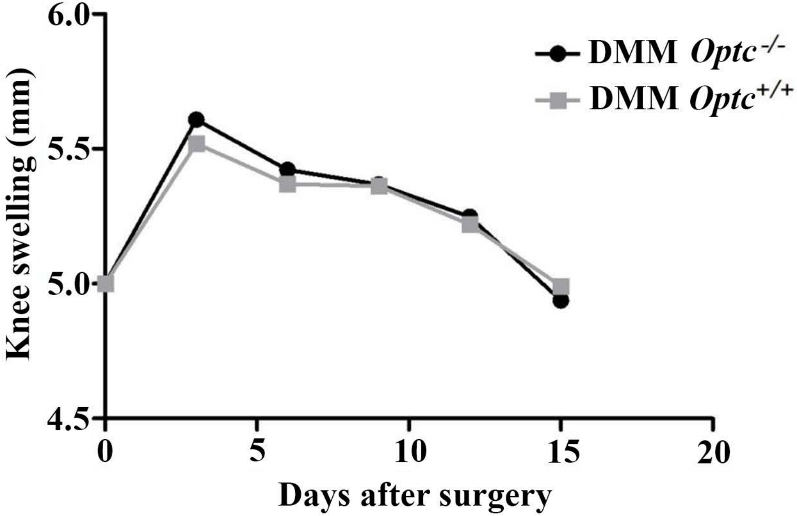

Supplementary Fig. S5. Knee joint swelling. Knee joint swelling was determined by the diameter (mm) of the DMM (right) knee in *Optc*$^{+/+}$ and *Optc*$^{-/-}$ mice (n=6). Mice were examined at baseline (day 0) and every 3 days following surgery until day 15 post-surgery. Data showed that the initial swelling (day 3 post-surgery) receded similarly in

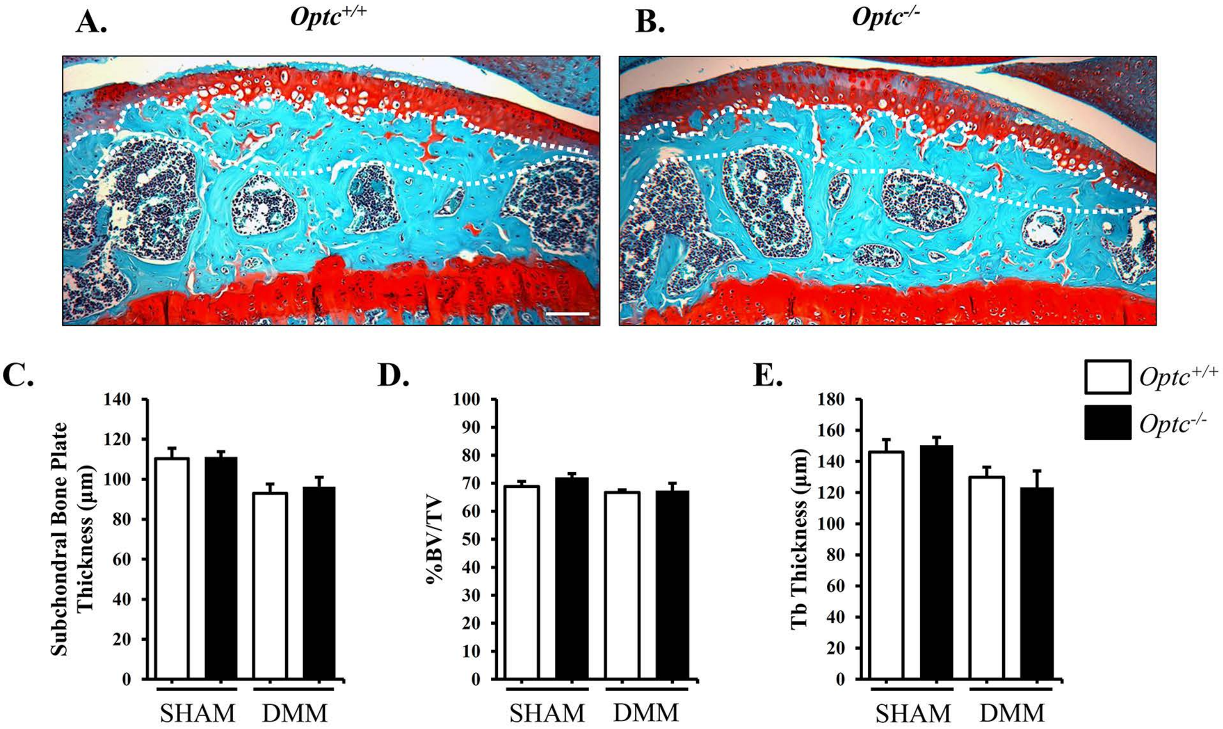

Supplementary Fig. S6. Histomorphometric analysis of the subchondral bone of the medial tibial plateau. (A, B) Representative sections of subchondral bone stained with Safranin-*O*/Fast green of **(A)** *Optc*$^{+/+}$ and **(B)** *Optc* $^{-/-}$ mice at 10 weeks after DMM surgery. White dotted lines in **A**, **B** delineate the subchondral bone plate thickness. Bar in **A** = 100 µm. **(C)** Subchondral bone plate thickness, **(D)** percentage of bone volume/total volume (%BV/TV) and **(E)** trabecular thickness (Tb Thickness) at 10 weeks post-surgery in sham (n=6/group) and DMM mice (*Optc*$^{+/+}$, n=9; *Optc*$^{-/-}$, n=8). Values are the mean±SEM. p values were determined by Mann-Whitney test and there was no statistically significant difference between *Optc*$^{+/+}$ and *Optc*$^{-/-}$ for sham or DMM.

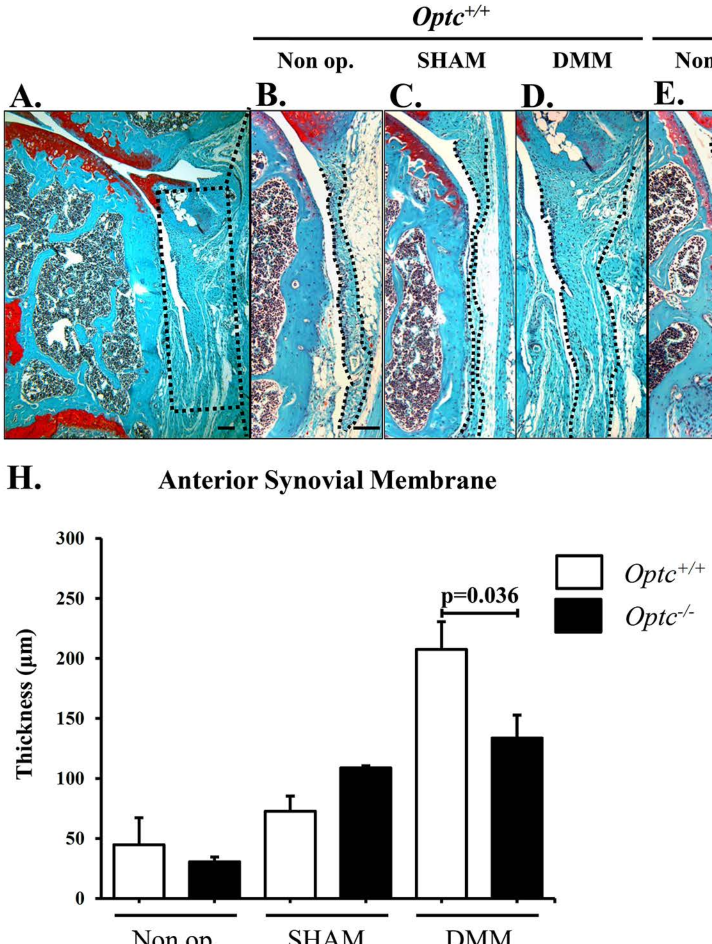

Supplementary Fig. S7. Synovial membrane evaluation. Representative sections of the anterior synovial membrane stained with Safranin-*O*/Fast green **(A-G)**. (**A)** Black boxed area represents the enlargement of **B-G**, and dotted lines in **B-G** the thickness of the synovial membrane. (**B**, **E)** *Optc*$^{+/+}$ and *Optc*$^{-/-}$ non-operated (Non op.) mice at 20 weeks old, and at 10 weeks after **(C**, **F)** sham (control) and (**D**, **G)** DMM surgery. Bars in **A** and **B** = 100 µm. (**H)** Histogram of the synovial membrane thickness of 22-week-old Non op. (*Optc*$^{+/+}$, n=4; *Optc*$^{-/-}$, n=7), sham (n=6/group) and DMM (*Optc*$^{+/+}$, n=9; *Optc*$^{-/-}$, n=8). Values are the mean±SEM. p values were determined by Mann-Whitney test; only significant p values are shown except those for *Optc*$^{+/+}$ between Non op./sham with DMM, and for *Optc*$^{-/-}$ between Non op. with sham and DMM, which are ≤0.017.

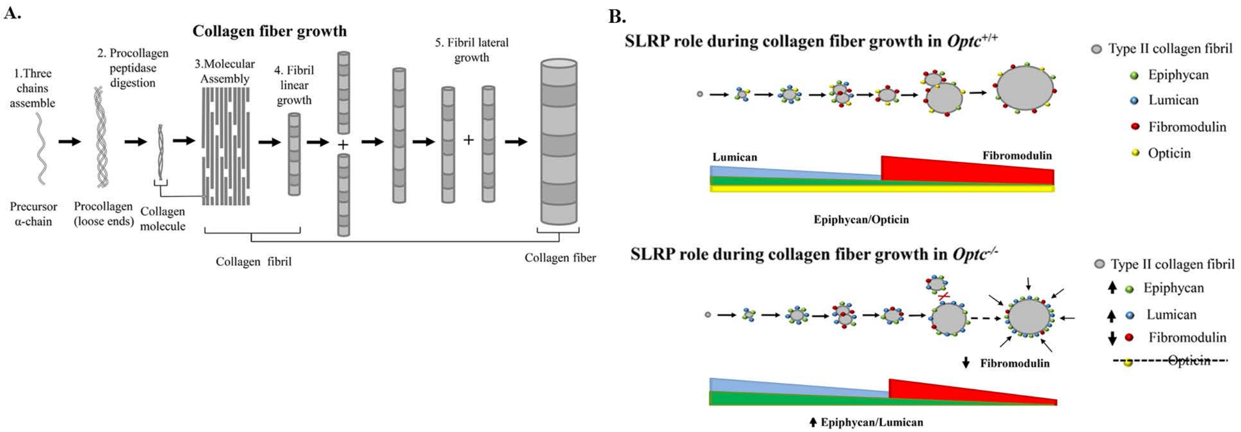

Supplementary Fig. S8. Representation of the collagen fibrillogenesis process. (A) During development stages, three precursor α-chain peptides assemble to form a procollagen molecule with loosened ends. The action of procollagen peptidase enzymes is essential for maturation of the collagen molecule. Several collagen molecules will covalently bond to other collagens in a scattered arrangement to form a collagen fibril. Fibril length grows by end-to-end fusion of several fibrils. At the end of the process, fibers grow in width by lateral fusion. **(B)** Involvement of SLRPs in the regulation of linear and lateral fibril growth. SLRPs bind to the fibril surface, regulating the linear and lateral growth of collagen fibrils during the fibrillogenesis process. In *Optc*$^{+/+}$ mice, lumican and fibromodulin combine their actions during the process by differential temporal expression. Lumican is involved in the first steps of the fibril formation, and as the development progresses in time, lumican expression decreases and fibromodulin takes place to promote the final growth steps. However, in *Optc*$^{-/-}$ there is a down-regulation of fibromodulin in adult mice leading to a compensating overexpression of lumican and therefore a domination of this molecule during the later steps of the fibril growth process. The abnormal excess of lumican in adult *Optc*$^{-/-}$ mice together with the overexpression of epiphycan result in the over coating of the collagen fibril preventing the rapid access of collagenases during osteoarthritis.

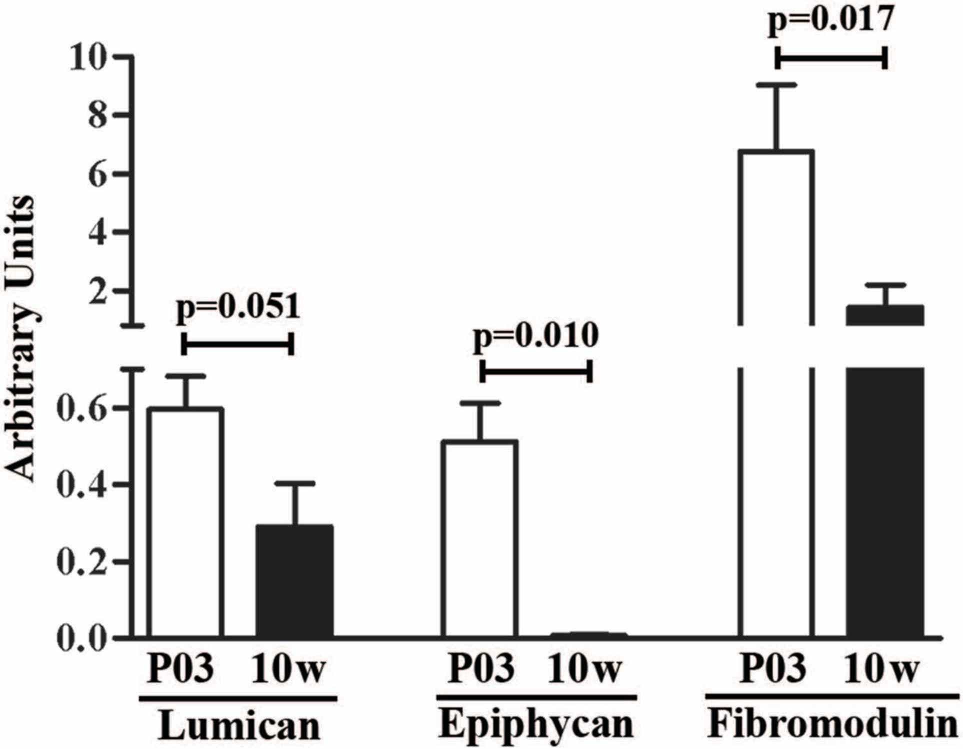

Supplementary Fig S9. SLRP expression in mouse cartilage. Lumican, epiphycan and fibromodulin mRNAs were extracted from articular cartilage of *Optc*$^{+/+}$ mice at day 3 (P03, n=6) and 10 weeks old (10w, n=5) and processed for qPCR. The value for each sample was calculated as the ratio of the number of molecules of the SLRP/the number of molecules of the housekeeping gene RPL19 and expressed as mean±SEM of arbitrary units. p values were determined by Mann-Whitney test.

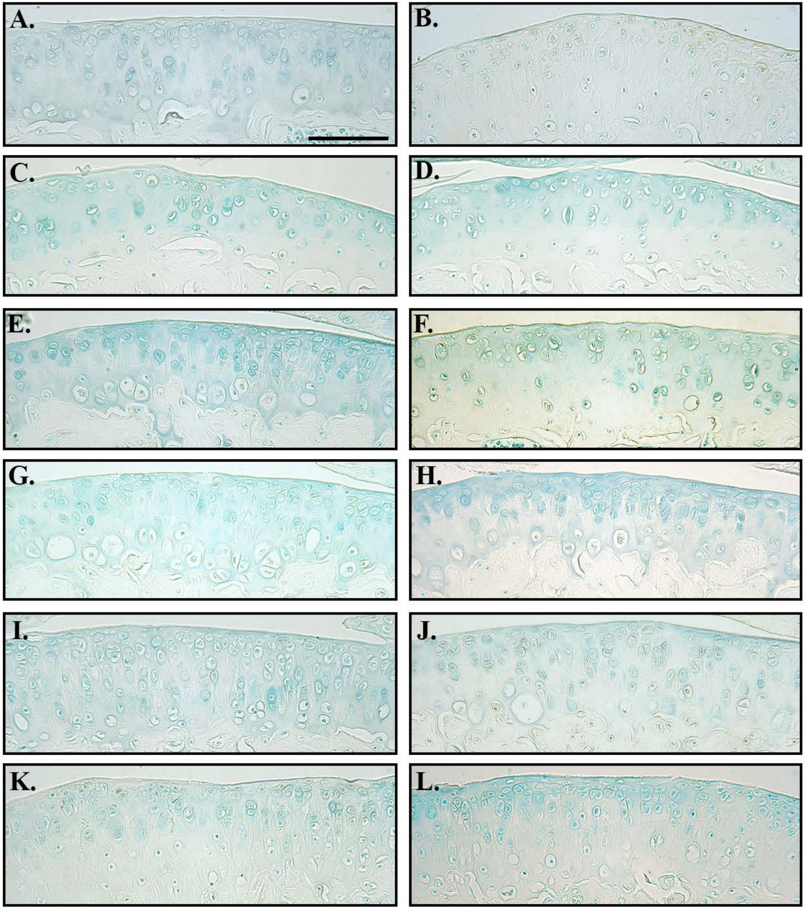

Supplementary Fig. S10. Immunohistochemistry negative controls. Control procedures for immunohistochemistry were performed on *Optc*$^{+/+}$ by substituting the primary antibody with a non-specific IgG, and in addition for type X collagen, by adsorption with the specific peptide. Illustrated are representative immunohistochemistry of non-operated mouse cartilage for **(A)** COL2-3/4Cshort, **(B)** VDIPEN, **(C)** type X collagen (non-specific IgG), **(D)** type X collagen (adsorption with the peptide), **(E)** MMP-13, **(F)** MMP-3, **(G)** lumican, **(H)** epiphycan, **(I)** fibromodulin, **(J)** C5b-9, **(K)** CCL2 and **(L)** opticin. These controls showed only background staining. Bar in **(A)** = 100 µm. Original magnification X250.

Supplementary Fig. S11. Full-length gel of electrophoresis separation of PCR products in Fig. S1.

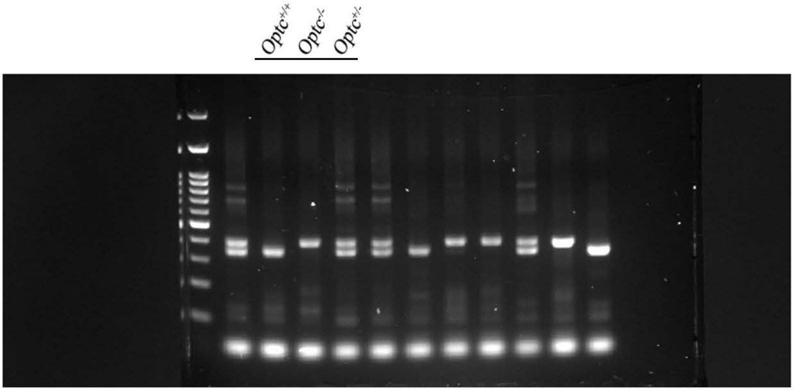

Acquisition Information

Imager	ChemiDoc™ XRS+
Exposure Time (sec)	11.789 (Auto - Intense Bands)
Flat Field	Applied (Lens)
Serial Number	721BR04040
Software Version	5.1
Application	SYBR Green
Excitation Source	UV Trans illumination
Emission Filter	Standard Filter

Image Information

Acquisition Date	2015-04-28 15:39:44
User Name	Farran Diaz-Cano Aina
Image Area (mm)	X: 133.8 Y: 100.0
Pixel Size (um)	X: 96.2 Y: 96.2
Data Range (Int)	6340 - 65535

Supplementary Fig. S12. Full-length gel of electrophoresis separation of PCR products in Fig S3.

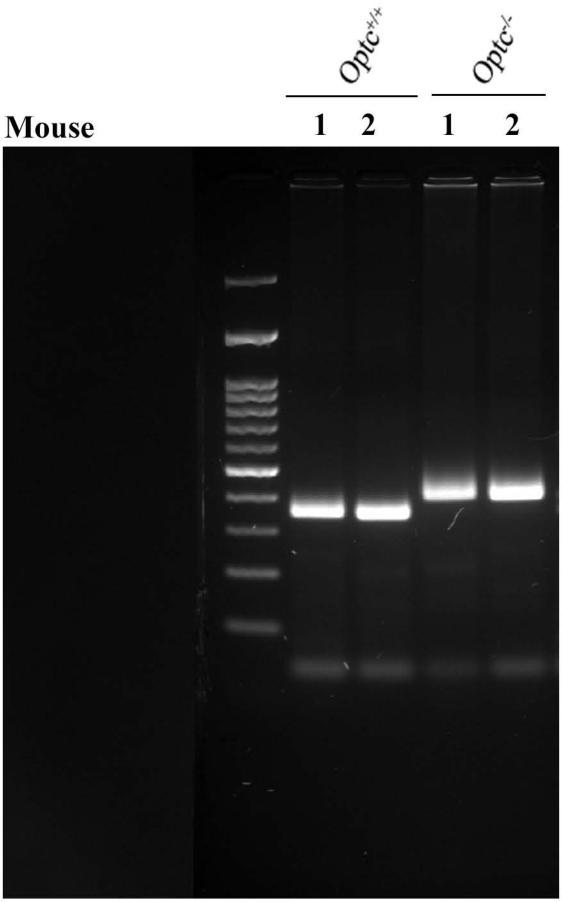

Acquisition Information

Imager	ChemiDoc™ XRS+
Exposure Time (sec)	5.516 (Auto - Intense Bands)
Flat Field	Applied (Lens)
Serial Number	721BR04040
Software Version	5.1
Application	SYBR Safe
Excitation Source	UV Trans illumination
Emission Filter	Standard Filter

Image Information

Acquisition Date	2016-11-11 11:31:42
User Name	Mineau François
Image Area (mm)	X: 115.0 Y: 85.9
Pixel Size (um)	X: 82.6 Y: 82.6
Data Range (Int)	5032 - 65535
